# Development and In Vivo Evaluation of a MGF110-1L Deletion Mutant in African Swine Fever Strain Georgia

**DOI:** 10.3390/v13020286

**Published:** 2021-02-12

**Authors:** Elizabeth Ramirez-Medina, Elizabeth Vuono, Sarah Pruitt, Ayushi Rai, Ediane Silva, Nallely Espinoza, James Zhu, Lauro Velazquez-Salinas, Manuel V. Borca, Douglas P. Gladue

**Affiliations:** 1 Plum Island Animal Disease Center, Agricultural Research Service, United States Department of Agriculture, Greenport, NY 11944, USA; Elizabeth.Ramirez@usda.gov (E.R.-M.); Elizabeth.Vuono@usda.gov (E.V.); Sarah.Pruitt@usda.gov (S.P.); ayushi.rai@usda.gov (A.R.); Ediane.Silva@usda.gov (E.S.); Nallely.Espinoza@usda.gov (N.E.); James.Zhu@usda.gov (J.Z.); Lauro.Velazquez@usda.gov (L.V.-S.); 2 Department of Pathobiology and Veterinary Science, University of Connecticut, Storrs, CT 06269, USA; 3 Department of Pathobiology and Population Medicine, Mississippi State University, P.O. Box 6100, Starkville, MS 39762, USA; 4 Oak Ridge Institute for Science and Education (ORISE), Oak Ridge, TN 37830, USA; 5 Department of Anatomy and Physiology, Kansas State University, Manhattan, KS 66506, USA

**Keywords:** African swine fever, African swine fever virus, ΔMGF110-1L

## Abstract

African swine fever (ASF) is currently causing an epizootic, affecting pigs throughout Eurasia, and causing significant economic losses in the swine industry. ASF is caused by African swine fever virus (ASFV) that consists of a large dsDNA genome that encodes for more than 160 genes; few of these genes have been studied in detail. ASFV contains four multi-gene family (MGF) groups of genes that have been implicated in regulating the immune response and host specificity; however, the individual roles of most of these genes have not been well studied. Here, we describe the evaluation of the previously uncharacterized ASFV MGF110-1L open reading frame (ORF) using a deletion mutant of the ASFV currently circulating throughout Eurasia. The recombinant ASFV lacking the MGF110-1L gene (ASFV-G-ΔMGF110-1L) demonstrated in vitro that the MGF110-1L gene is non-essential, since ASFV-G-ΔMGF110-1L had similar replication kinetics in primary swine macrophage cell cultures when compared to parental highly virulent field isolate Georgia2007 (ASFV-G). Experimental infection of domestic pigs with ASFV-G-ΔMGF110-1L produced a clinical disease similar to that caused by the parental ASFV-G, confirming that deletion of the MGF110-1L gene from the ASFV genome does not affect viral virulence.

## 1. Introduction

Until recently, African swine fever (ASF) was historically endemic in Africa and Sardinia (Italy). In 2007, a highly virulent strain of African swine fever virus (ASFV), ASFV strain Georgia (ASFV-G), was introduced into the Republic of Georgia, quickly spreading into neighboring countries throughout Eastern Europe [1]. Then, in 2018, ASF was introduced into China, where the disease quickly spread across Southeast Asia. In 2020, ASF was first detected in Germany, although as of this publication it has been detected only in the wild boar population. This continued seemingly uncontrolled spread of ASFV has the potential to cause a worldwide protein availability shortage, as well as large economic losses in the swine industry [2].

ASF is a frequently lethal viral disease of swine caused by a large dsDNA virus (180–190 kilobase pairs), known as ASFV. Depending on the viral isolate, the viral infection can produce various clinical presentations in domestic pigs, from being sub-clinical to highly lethal [3]. Currently, there is no commercial vaccine available for ASF. To control outbreaks, susceptible animals in infected farms are culled, and strict biosecurity measures are implemented to prevent the introduction of ASFV onto uninfected farms. ASFV experimental vaccines have shown promise against the current circulating strain in Europe and Asia; these vaccines are live attenuated viruses containing the deletion of one or more proteins from contemporary field isolates [4,5,6,7,8,9]. It is important to understand the role of individual genes in ASFV, as well as how their possible manipulation could be used to develop experimental vaccines, and/or to have the tools necessary to develop the next generation of ASF vaccines.

ASFV has more than 160 open reading frames (ORFs) [1]. Few of these ORFs have been experimentally characterized, with their predicted functional role based solely on sequence homology to previously investigated proteins. The role of specific ASFV genes involved in viral virulence is largely unknown [3]. The identification of viral proteins that are important for in vitro and/or in vivo virus replication or virus virulence is critical information needed to develop novel antivirals or vaccines for disease control. The discovery of ASFV gene function using genetic manipulation techniques has resulted in several experimental ASFV live-attenuated vaccines [4,5,8,10,11]. Only a small number of virus genes have been successfully deleted from the ASFV genome, producing novel deletion mutants of the virus (e.g., TK, NL, CD2, MGF360-16R, MGF360-1L, L83L, C962R and X69R) [12,13,14,15,16,17,18], as well as another small number of genes determined to be essential for virus replication (e.g., EP152R, p30, p54 and p72) [19,20,21,22]. The absence of this experimental information greatly restricts the knowledge for most ASFV proteins only to predicted functions by functional genomics.

The MGF110 family consists of thirteen highly diverse paralogs among genomes [23]. The ASFV-G genome contains 11 different MGF110 family genes (1L, 2L,3L,4L, 5L-6L, 7L, 9L, 10-14L, 12L, 13La and 13Lb) all contained in the left terminal repeat region. Fusion(s) between some MGF110 proteins and frameshift mutations vary among ASFV genomes; the ASFV-G genome contains a fusion of the MGF110 5L and 6L proteins, as well as the 10L and 14L proteins. The ASFV-G ORF 13L has a frameshift mutation that splits the ORF into 13La and 13Lb genes. Interestingly, the only MGF110 gene that is present in all ASFV genomes is MGF110-1L, suggesting that MGF110-1L is necessary for ASFV survival. This study aims to investigate the role of the previously uncharacterized MGF110-1L.

## 2. Materials and Methods

### 2.1. Viruses and Cells

Primary swine macrophage cell cultures were prepared from defibrinated swine blood, as previously described [24]. Swine blood was treated with heparin and incubated at 37 °C for 1 h to allow erythrocyte separation. Mononuclear leukocytes were purified by a Ficoll-Paque (Pharmacia, Piscataway, NJ, USA) density gradient (specific gravity, 1.079). The monocyte/macrophage cell fraction was cultured for 48 h at 37 °C under 5% CO2.

Adherent cells were detached from the Primaria flasks and reseeded into Primaria T25, 6- or 96-well dishes at a density of 5 ×10^6^ cells per ml for use in assays 24 h later. ASFV Georgia (ASFV-G) was a field isolate kindly provided by Dr. Nino Vepkhvadze, from the Laboratory of the Ministry of Agriculture (LMA) in Tbilisi, Republic of Georgia [10].

Comparative growth curves between ASFV-G-ΔMGF110-1L and parental ASFV-G were performed in primary swine macrophage cell cultures in 24-well plates, and infected at an MOI of 0.1 (based on HAD50 (50% hemadsorbing doses), as previously determined in primary swine macrophage cell cultures). After 1 hour of adsorption at 37 °C under 5% CO2 the inoculum was removed, and the cells were rinsed two times with PBS. The monolayers were then rinsed with macrophage media and incubated for 2, 24, 48, 72 and 96 h at 37 °C under 5% CO2. At appropriate times post-infection, the cells were frozen at ≤221270 °C, and the thawed lysates were used to determine titers by HAD50/mL in primary swine macrophage cell cultures. All samples were run simultaneously to avoid inter-assay variability. Virus titration was performed on primary swine macrophage cell cultures in 96-well plates. Virus dilutions and cultures were performed using macrophage medium. The presence of the virus was assessed by hemadsorption (HA), and virus titers were calculated as previously described [25].

### 2.2. Construction of the MGF110-1L Deletion Mutant ASFV

**Figure 1:**
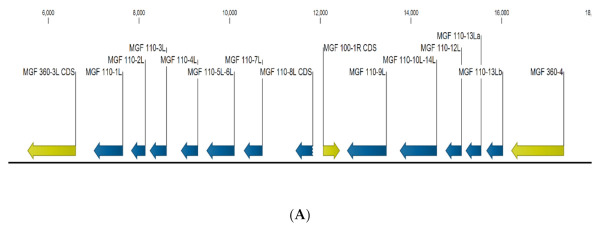

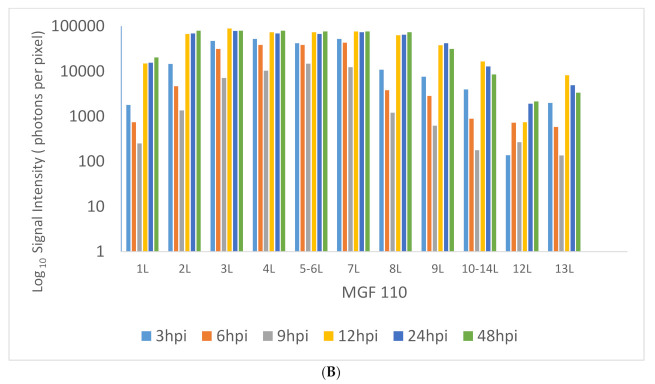
(**A**) Location and orientation of all MGF110 family of genes. The MGF110 genes are depicted in Blue. Other multi-gene family (MGF) genes within this region of the ASFV-G genome are depicted in yellow (**B**) Expression levels of individual MGF 110 genes at the indicated timepoints, expression is measured in Log_10_ Signal Intensity.

Recombinant ASFV-G-ΔMGF110-1L was generated by homologous recombination between the parental ASFV genome and a recombination transfer vector, following previously described procedures [6]. The recombinant transfer vector (p72mCherryΔMGF110-1L) contained flanking genomic regions: the left arm is located between genomic positions 6003–7003, and the right arm is located between genomic positions 7649–8649 and a reporter gene cassette containing the mCherry fluorescent protein (mCherry) gene under the control of the ASFV p72 late gene promoter [26]. The recombinant transfer vector was obtained by DNA synthesis (Epoch Life Sciences, Sugar Land, TX, USA). This construction created a 645-nucleotide deletion between nucleotide positions 7004-7648, deleting the complete ORF sequence of MGF110-1L (Figure 1). Macrophage cell cultures were infected with ASFV-G and transfected with p72mCherryΔMGF110-1L. The deletion mutant ASFV-G-ΔMGF110-1L was purified to homogeneity by successive rounds of limiting dilution purification, using the highest dilution with detectable amounts of mCherry. ASFV DNA was extracted from infected cells, and a full-length sequence using next-generation sequencing (NGS) was obtained, as described previously [26], using an Illumina NextSeq500 sequencer with standard sequencing protocols. Analysis of the sequence was done using CLC Genomics Workbench software version 20 (QIAGEN, Hilden, Germany).

### 2.3. Animal Experiments

ASFV-G-ΔMGF110-1L was assessed for its virulence phenotype relative to the parental ASFV-G virus using 36-41-lilogram commercial breed swine. Five pigs were inoculated intramuscularly (IM) with 10^2^ HAD_50_ of ASFV-G-ΔMGF110-1L, and compared with a group of pigs inoculated with similar doses of ASFV-G. Clinical signs (anorexia, depression, fever, purple skin discoloration, staggering gait, diarrhea and coughs) and changes in body temperature were recorded daily throughout the experiment. Animal experiments were performed under biosafety level 3 conditions in the animal facilities at Plum Island Animal Disease Center, following a strict protocol approved by the Institutional Animal Care and Use Committee (225.01-16-R_090716)

## 3. Results and Discussion

### 3.1. Expression and Location of MGF110 Family of Genes

The MGF110 family of genes in ASFV-G is located in the Left Variable region between nucleotide positions 7004 and 16031. This 9kb region contains all 110 genes and one other multi-gene family (MGF) gene, MGF100-1R (Figure 1A). The transcriptional activity of the MGF110 family of genes during the infectious cycle was analyzed using deposited microarray data from a previous study [27]. Transcription was detected for all MGF genes at all time points, increasing throughout the experiment, with a dip in expression levels similar to what has been observed with early gene p30. Interestingly, MGF110-8L was detected despite not having a traditional start codon, suggesting that an alternative start site was able to drive the expression of this ORF.

The presence of the MGF110 family RNA transcripts was detected by DNA microarray analysis, as we have previously reported, and are available in the GEO repository under the series record GPL26793 [6]. All MGF110 family of transcripts were detected at all time points. Expression gradually decreased from 3 to 9 hpi, then increased at 12 to 18 hpi. The transcriptional pattern was similar to the early protein p30 (CP204L) as previously reported [3]. Results demonstrated that the ASFV I8L gene encodes for a protein that is abundantly expressed early in the virus replication cycle.

### 3.2. MGF110-1L Gene is Conserved across Different ASFV Isolates and Transcribed as an Early Viral Gene

**Figure 2:**
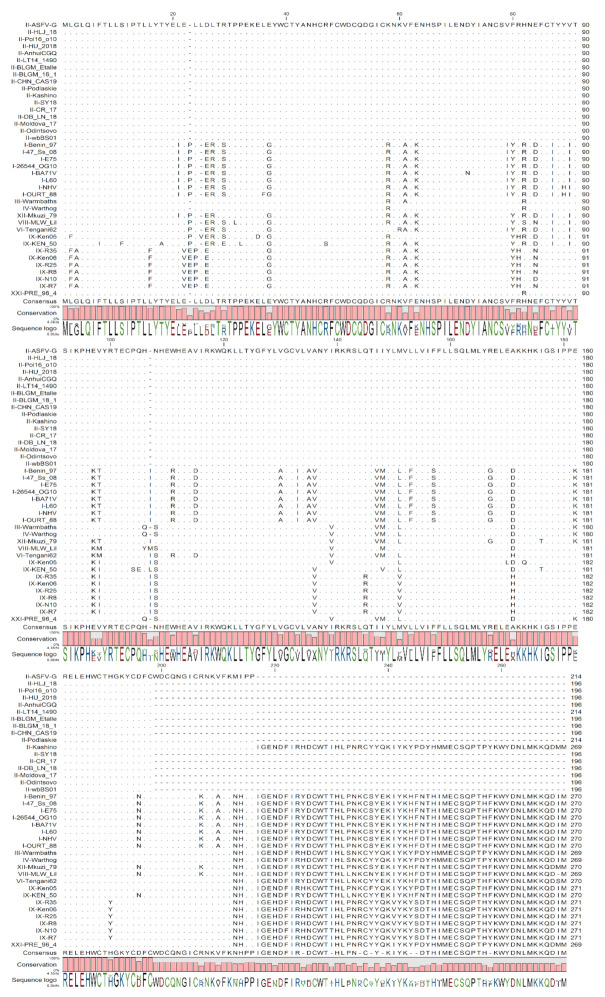
Multiple sequence alignment of the indicated African swine fever virus (ASFV) isolates of viral protein MGF110-1L. Matching residues are represented as dots. The degree of conservation is below the alignment.

MGF110-1L is the only MGF gene in family 110 that is present in all ASFV genomes (Figure 2). Multiple sequence alignments across all published isolates of ASFV were performed using CLC Genomics Workbench, revealing that MGF110-1L proteins are 196 to 271 amino acids in length. The difference in length appears to be due to the larger proteins having an extension on the C-terminus. The protein sequence appears to be highly conserved when comparing MGF110-1L proteins of similar length, or overlapping partial regions of the protein from different isolates. Particularly, when the ASFV genotype II sequences are compared, MGF110-1L proteins are highly conserved with 100% amino acid identity, for the first 196 amino acids, isolates with longer MGF110-1L, such as ASFV-G with a 214 amino acid protein is 100% conserved with genotype II sequences that contain another 214 amino acid protein one genotype II isolate, Kashino has a 269 amino acids protein. Furthermore, any non-conserved residues of other genotypes appear to be mostly conserved when compared within their associated genotype, with an overall conservation of all isolates containing 84% of all residues in the ASFV-G protein sequence.

### 3.3. Development of the ASFV-G-ΔMGF110-1L Deletion Mutant

**Figure 3:**
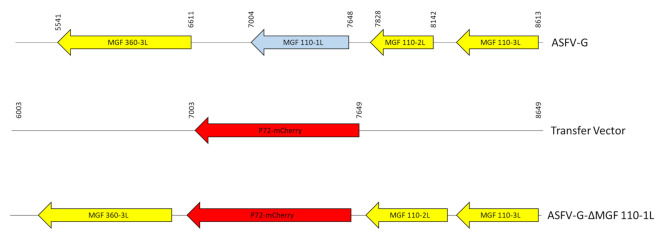
Schematic for the development of ASFV-G-ΔMGF110-1L. The transfer vector contains the p72 promoter and a mCherry cassette; the flanking left and right arms are indicated, and were designed to have flanking ends to both sides of the deletion/insertion cassette. The resulting ASFV-G-ΔMGF110-1L virus with the cassette inserted is shown on the bottom.

To study the function of the MGF110-1L gene during ASFV replication in cell cultures and the process of virulence in swine, a MGF110-1L deletion mutant in ASFV-G was developed (ASFV-G-ΔMGF110-1L) (Figure 3). Deletion of MGF110-1L was achieved by substituting the complete ORF of 214 amino acid residues of the MGF110-1L with a p72mCherry cassette following standard methodologies based on homologous recombination [12]. ASFV-G-ΔMGF110-1L was constructed from the highly virulent ASFV Georgia 2007 isolate (ASFV-G). A region spanning 645-bp (between nucleotide positions 7004 and 7648) was deleted from the ASFV-G genome and substituted with a 1226-bp cassette containing the p72mCherry construct (see Material and Methods). ASFV-G-ΔMGF110-1L was obtained after a process of successive limiting dilution purifications in swine macrophage cell cultures. The virus obtained from the last purification round was further amplified in primary swine macrophage cell cultures to obtain a virus stock.

The accuracy of the genetic modifications introduced into ASFV-G-ΔMGF110-1L, as well as the conservation of the integrity of the remaining virus genome was assessed by full genome sequence obtained by NGS on an Illumina NextSeq^®^ 500. The comparison between ASFV-G-ΔMGF110-1L and ASFV-G full-length genomes demonstrated a deletion of 645 nucleotides, which is consistent with the introduced modifications. Furthermore, the ASFV-G-ΔMGF110-1L genome contained a 1226-nucleotide insertion corresponding to the introduction of the p72-mCherry cassette sequence. No additional significant differences were observed between the ASFV-G-ΔMGF110-1L and ASFV-G genomes, confirming that the ASFV-G-ΔMGF110-1L virus did not acquire additional mutations during the process of homologous recombination, or the limiting dilution purification procedure. NGS results also demonstrated the absence of any residual parental ASFV-G genome as a potential contaminant of the ASFV-G-ΔMGF110-1L stock.

### 3.4. Replication of ASFV-G-ΔC962R in Primary Swine Macrophages

**Figure 4:**
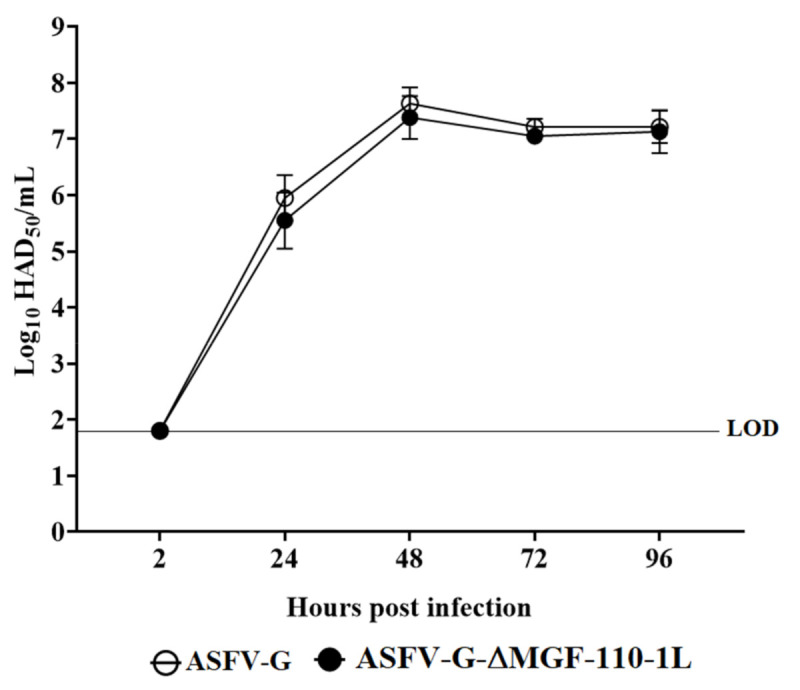
In vitro growth kinetics in primary swine macrophage cell cultures for ASFV-G-Δ MGF110-1L and parental ASFV-G, infected (MOI = 0.01) with ASFV-G-Δ MGF110-1L or parental ASFV-G viruses. Samples were taken from three independent experiments at the indicated time points and titrated. Data represent means and standard deviations. Sensitivity using this methodology for detecting virus is >log10 1.8 HAD_50_/mL. No significant differences in viral yields between viruses were observed at any time point tested determined using the Holm-Sidak method (α = 0.05), without assuming a consistent standard deviation. All calculations were conducted using the software Graphpad Prism version 8.

**Figure 5:**
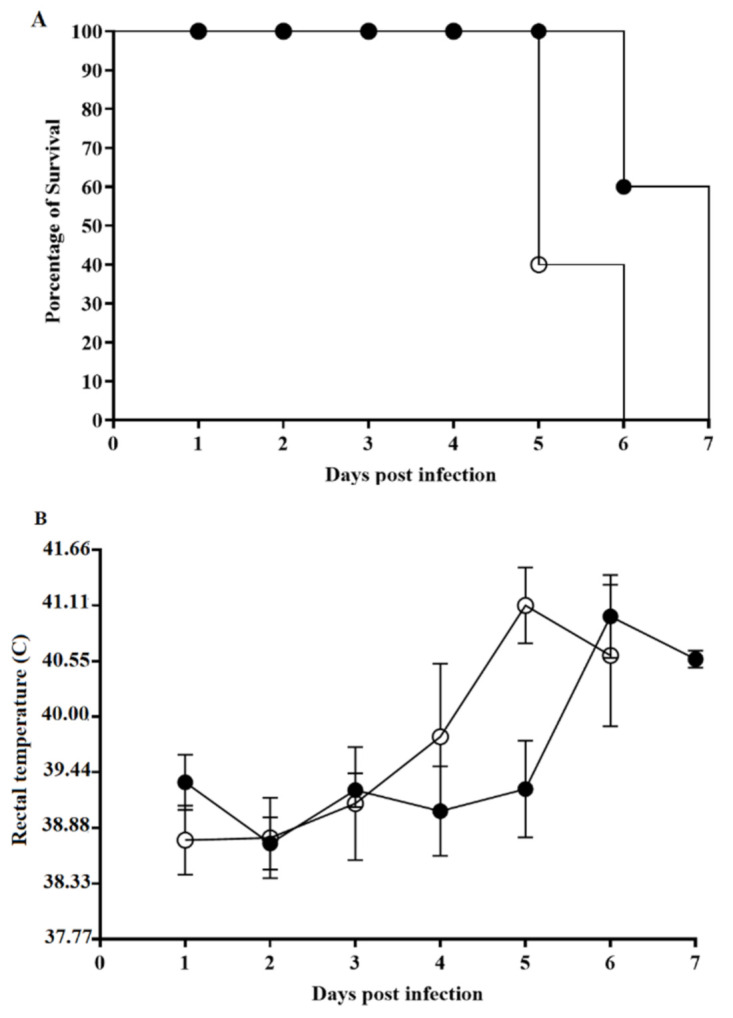
Evolution of mortality (**A**) and body temperature (**B**) in animals (5 animals/group) intramuscularly (IM) infected with 10^2^ HAD_50_ of either ASFV-G-ΔMGF110-1L (filled symbols) or parental ASFV-G (open symbols). Significant differences (*p*-value = 0.0201) in the survival course between groups of pigs were found using the test de Long-rank (Mantel-cox test). No statistical differences were found in body temperatures between pigs in both groups, when evaluated by the Holm-Sidak method (α = 0.05). All calculations were conducted using the software Graphpad Prism version 8.

To assess the function of MGF110-1L during virus replication, the in vitro growth kinetics of ASFV-G-ΔMGF110-1L were evaluated in swine macrophage cultures (Figure 4). ASFV-G-ΔMGF110-1L kinetics were compared to that of the parental ASFV-G in a multistep growth curve. Macrophage cultures were infected with either virus at a MOI of 0.01 and samples were collected at 2, 24, 48, 72 and 96 hpi. Results demonstrated that ASFV-G-ΔMGF110-1L displayed a similar growth kinetic when compared to the parental ASFV-G (Figure 5). Thus, deletion of the MGF110-1L gene does not significantly affect the ability of ASFV-G to replicate in swine macrophages. This was surprising, considering that all genomes of ASFV contain MGF110-1L. However, it is possible that the function of MGF110-1L could be duplicated by other MGF110 genes, or another gene in ASFV.

### 3.5. Assessment of ASFV MGF110-1L Virulence in Swine

To assess the consequences of the deletion of the MGF110-1L gene to ASFV-G virulence, a group (*n* = 5) of 36–41 kilogram pigs were IM inoculated with 10^2^ HAD_50_ per animal, while an additional control group was also IM inoculated with 10^2^ HAD_50_ of the parental ASFV-G. As expected, animals inoculated with virulent ASFV-G had an increase in body temperature (>40 °C) by day 4–5 pi, which was followed by the quick appearance of ASF-associated clinical signs (including anorexia, depression, purple skin discoloration, staggering gai, and diarrhea) (Table 1 and Figure 5).

Signs of the disease rapidly aggravated, and animals were euthanized in extremis by day 5–6 pi. Animals receiving ASFV-G-ΔMGF1101L presented with a clinical disease very similar to that described for animals infected with ASFV-G. Both the time of presentation and severity of the clinical signs resembled those present in animals inoculated with the parental virus. Consequently, deletion of the MGF110-1L gene does not alter the virulence phenotype of the highly virulent ASFV-G isolate.

**Figure 6:**
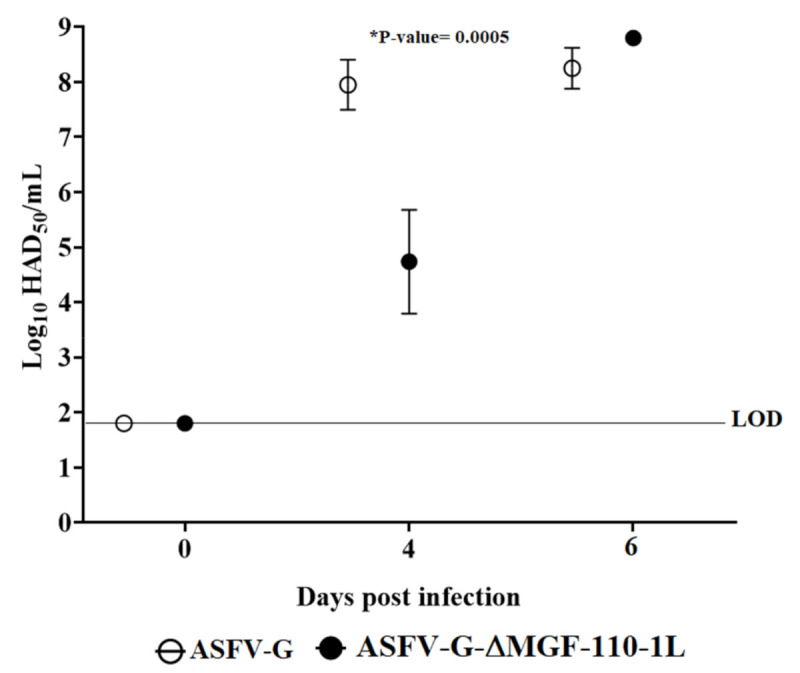
Viremia titers detected in pigs IM inoculated with 10^2^ HAD_50_ of either ASFV-G-ΔMGF110-1L (filled symbols) or ASFV-G (empty symbols). Each symbol represents the average of animal titers in each of the groups. Sensitivity of virus detection: >log10 1.8 TCID_50_/mL. Significant differences in viremia values between both groups of pigs were found at day four post-infection using the Holm-Sidak method (α = 0.05) without assuming a consistent standard deviation. All calculations were conducted on the software Graphpad Prism version 8.

The analysis of viremias in animals IM infected with parental ASFV-G demonstrated expected high titers (10^6^–10^7.85^ HAD_50_/mL) on day 4 pi, remaining similarly high by day 7 pi, when all animals were euthanized. Animals inoculated with ASFV-G-ΔMGF110-1L presented with viremia values ranging from 10^6^–10^6.8^ HAD_50_/mL by day 4 pi, reaching similar titers of those animals infected with ASFV-G by day 7 pi, which was also the last sample time taken before animals were humanely euthanized (Figure 6). The presentation of these indistinguishable clinical signs suggests that the MGF110-1L gene is not required for ASFV virulence.

In summary, we determined that MGF110-1L, the only MGF110 family gene, is present in all sequenced isolates, is a highly conserved protein among ASFV isolates [20], is an early protein that is a non-essential gene since its deletion from the ASFV-G genome does not significantly alter virus replication in swine macrophage cultures, and importantly, is not critical for ASFV virulence in swine, as the deletion mutant ASFV-G-ΔMGF110-1L had similar pathogenesis as the parental ASFV-G.

## Figures and Tables

**Table 1:** Swine survival and fever response following infection with ASFV-G-ΔMGF110-1L and parental ASFV-G.

			Fever		
Virus (10^2^ HAD_50_)	No. of Survivors/Total	Mean Time to Death (±SD)	No. of Days to Onset (±SD)	Duration No. of Days (±SD)	Maximum Daily Temp, °C (±SD)
ASFV-G-ΔMGF110-1L	0/5	6.6 (0.55)	6 (0.0)	0.6 (0.55)	41.1(0.41)
ASFV-G	0/5	5.4 (0.55)	4.4 (0.55)	1 (1)	41.1 (0.38)

## Data Availability

Data is contained within the article.
